# Prevalence and determinants of smoking status among university students: Artvin Çoruh University sample

**DOI:** 10.1371/journal.pone.0200671

**Published:** 2018-12-10

**Authors:** Dilek Karadoğan, Özgür Önal, Yalçın Kanbay

**Affiliations:** 1 Department on Chest Diseases, Recep Tayyip Erdoğan University, School of Medicine, Rize, Turkey; 2 Department of Public Health, Süleyman Demirel University, School of Medicine, Isparta, Turkey; 3 Department of Psychiatric Nursing, School of Health Science, Çoruh University, Artvin, Turkey; National Institute of Health, ITALY

## Abstract

**Background and aim:**

Smoking is still a public health concern in many countries, especially among young adults. Consequently, we determined what factors affect university students’ smoking behavior in Turkey.

**Methods:**

This cross-sectional study was conducted between March and June 2017 using a simple random sampling method. A self-administered questionnaire was used to collect information on participants’ (N = 2,505; mean age = 20.9 ± 2.5 years; 58.9% women) sociodemographic characteristics, cigarette smoking status, and related risk factors. Univariate analysis and multivariate logistic regression analysis were performed with the Backward likelihood-ratio method.

**Results:**

Students were completing either two- or four-year degrees (45.6% and 54.4%, respectively). Regarding familial smoking behavior, 36.1% had a father who smoked, 10.3% had a mother who smoked, and 15.0% had siblings who smoked. Among participants, 27.9% were current smokers: 46% of the men and 15.3% of the women. Mean smoking onset age was 16.34 ± 2.72 years (15.65 ± 2.67 years for men and 16.34 ± 2.72 for women (p < .05). Mean Fagerströmtest score was 4.43 ± 1.82, and women had lower test scores than did men (p < .05). After controlling for potential confounders in multivariate analyses, five factors were significantly positively associated with current smoking: being a man (odds ratio (OR): 3.43; 95% confidence interval (CI): 2.75–4.28), studying in a two-year program (OR: 1.74; 95% CI: 1.39–2.18), having at least one immediate family member who smoked (OR: 1.63; 95% CI: 1.31–2.04), having all close friends who smoked (OR: 1.81; 95% CI: 1.40–2.33), and alcohol consumption (OR: 4.39; 95% CI: 3.51–5.49).

**Conclusion:**

There was a higher smoking rate among our study population, both compared to similar national studies and Turkey’s overall smoking rate. Underlying factors should be evaluated via qualitative studies and preventive strategies should be implemented accordingly.

## Introduction

Cigarettes are the most commonly used form of tobacco and one of the major causes of preventable diseases globally [1]. Turkey’s fight against tobacco depends on following the evidence-based tobacco control measures and policies that have been identified as effective by the World Health Organization (WHO) [2]. Turkey protects its population with five tobacco control measures, known as “MPOWER” policies [3, 4]. Turkey’s smoking prevalence among individuals aged older than 15 years was 44.5% in 1988; however, after anti-tobacco policies were implemented, that rate decreased to 33.6% in 1993. In 2004, Turkey signed the Framework Convention on Tobacco Control with the WHO, and, by 2008, the smoking rate for that population decreased slightly to 31.2% [4]. A recent study explained that, despite the strict anti-tobacco policies and decreased legal tobacco product sales, as of 2006, the Turkish current smoking rate had plateaued after a 20-year decline. However, this still leaves Turkey with a higher smoking rate than other countries, such as the United States [5]. According to the last Global Adult Tobacco Survey, that rate finally decreased to 27.1% in 2012, and the MPOWER policies are the main factor in that result [4]. Among the studies that isolated university students’ smoking rates, the situations were similar to the general population. After the implementation of MPOWER policies, researchers also found a decrease in smoking prevalence among university students from 2005–2006 to 2012–2013: 26.9% to 18.5% [6].

In general, the smoking rates in Turkey differ nationwide according to socioeconomic status. A national study found no difference before and after the tobacco control policies in the poorest population, while the smoking rate did decrease among the richest population [3]. The continued levels of smoking rates in the poorest population, despite decreased legal cigarette sales, were attributed to illegal tobacco use, either as cigarettes or in other forms [3]. In a recent study, Turkey’s overall illicit cigarette use was determined to be around 12%, most commonly observed in the eastern part of the country [7]. Therefore, prevalence studies should be conducted periodically and should evaluate regional data.

Youth are the main target for tobacco companies, and the age of starting to smoke is decreasing in developing countries. Smoking for the first time before the age of 18 years contributes to life-long smoking, and, for those smokers, quitting is more difficult [8]. Universities are a key reflection of the general data on the younger population’s smoking behaviors. In Turkey, university students traditionally leave their homes to move to another city and start living with their peers; therefore, the decreased family pressure and control allows students more freedom to make their own decisions. Consequently, university life itself can be a risk factor for both the initiation of and increases in smoking behavior. Accordingly, studies that compare first- and last-year university students’ smoking levels [9, 10]—and studies that follow Turkish students prospectively—revealed an increased smoking rate during university life [11]. Preventative measures during this period are critical, such as campus-wide smoking bans, educational symposiums about the harms of tobacco, and other measures that are known to negatively impact smoking rates [12, 13].

The factors associated with smoking among university students have been evaluated by previous national and international studies. The primary factors associated with student smoking status include socioeconomic status, family/friends’ smoking behavior, alcohol use, sex, faculty, year of education, and residing with friends [14, 15]. However, diverse geographical regions have different risk factors due to their cultural-sociological differences; therefore, there is a need to conduct studies to evaluate distinct regions’ statuses.

Our setting for this study was a university located in the Eastern Black Sea region of Turkey. It does not have any smoking bans on campus, and the students are mostly from the eastern and north eastern parts of the country. Students were mostly from low- and middle-income families. As mentioned above, these areas of the country have the highest illicit tobacco use in Turkey, both thin-paper rolled cigars and cigarettes. Further, the city where the university is located shares a border with Georgia and is involved in that country’s illicit tobacco transport [16].

We evaluated the current smoking prevalence of Artvin Çoruh University students and the influencing factors on their current smoking status, which is critical to elucidate the smoking trends in the eastern part of Turkey. This study provides data that assist in strengthening policies against smoking and ultimately aid universities in developing anti-smoking programs.

## Materials and methods

### Setting and study design

This cross-sectional study was conducted at Artvin Çoruh University. Artvin is a small city in the Eastern Black Sea region of Turkey and shares a border with Georgia. We included participants from nine different schools or faculties within the university—Faculty of Education, Faculty of Art and Sciences, Faculty of Economics and Administrative Sciences, Faculty of Management, Faculty of Health Sciences, Faculty of Forestry, Faculty of Engineering, Vocational School, and the vocational high school—from March 1 to June 30, 2017.

Artvin Çoruh University had approximately 9,000 students in the 2016/2017 educational year; at the time of data collection, there were 6,583 students in those departments. Based on the assumption of a margin of error of 2% and a confidence interval of 99%, the initial/minimum sample size was calculated to be 2,549 students. The investigators ultimately decided to distribute the survey to 2,741 students, accounting for an expected number of nonresponses and incomplete questionnaires, by using a simple random sampling method. After excluding any incomplete and unsound responses, we utilized data from 2,505 students.

This research was approved by Artvin Çoruh University Ethical Committee and by the Vice Rector of Çoruh University.

### Data collection

We employed a short, self-administered questionnaire. The questionnaire was succinct to encourage students to respond. It comprised 26 questions that were modified from previously used surveys in Turkey [15, 17, 18], including 12 questions about respondents’ socioeconomic profile and 8 questions about their attitude toward smoking and alcohol. The last 6 questions, the Fagerström test [19], were for the regular smokers. A smoker in this study was defined as a participant who had smoked regularly in the last 30 days prior to completing the questionnaire and had smoked at least 100 cigarettes in his/her lifetime. A non-smoker was defined as someone who did not smoke in the previous 30 days and/or had not smoked 100 cigarettes in his/her lifetime or had smoked over 100 cigarettes in their his/her but none in last 30 days [20]. A team of 10 assistants was trained to ensure a unified procedure for data collection. A preliminary pilot study was undertaken with 50 students and some questions were subsequently rephrased and modified accordingly.

### Statistical analyses

Data were entered and analyzed using SPSS version 20.0. Descriptive statistics and a logistic regression analysis were performed. For categorical variables, Pearson’s chi-square test was used; for continual variables students’ t-test was used. A multivariate logistic regression analysis using the backward likelihood-ratio method was conducted to identify factors independently associated with students’ smoking status. P-values < .05 were considered significant.

## Results

Overall, 2,505 students’ data were evaluated: 58.9% were women and 41.1% were men. Students’ mean age was 20.87 ± 2.50 years. The overall smoking rate was 27.9%: 15.9% of the women and 46.0% of the men. Detailed demographical characteristics of the study population are shown in Tables 1 and 2.

**Table 1:** Sociodemographic characteristics of the students according to smoking status (categorical variables).

	Smoker (699, %27.9) n(%row)	Total (2505, %100.0) n(%column)	p
**Gender**			
Female	226 (15.3)	1476 (58.9)	**<0.001**
Male	473(46.0)	1029 (41.1)	
**Consumption of alcohol**			
**Present**	343 (59.7)	575 (23.0)	**<0.001**
**Absent**	356 (18.4)	1930 (77.0)	
**At least one smoker family member**			
**Present**	492 (32.0)	1538 (61.4)	**<0.001**
**Absent**	207 (21.4)	967 (38.6)	
**Smoker close friends**			
**Some**	472 (22.8)	2074 (82.8)	**<0.001**
**All**	227 (32.5)	431 (17.2)	
**Curricula**			
**4 years faculties**	283 (20.8)	1363 (54.4)	**<0.001**
**2 years faculties**	416 (36.4)	1142 (45.6)	
**Healthcare related faculty student**			
**Yes**	142(22.4)	634 (25.3)	**<0.001**
**No**	557 (29.8)	1871 (74.7)	
**Class**			
**1**	324 (29.0)	1119 (44.7)	0.193
**2**	251 (28.3)	888 (35.4)	
**3**	55 (22.2)	248 (9.9)	
**4**	69 (27.6)	250 (10.0)	
**Family type**			
**Intact**	32 (47.8)	67 (2.7)	**<0.001**
**Seperated**	667 (27.4)	2438 (97.3)	
**Mother’s education level**			
**≤5 years**	480 (26.3)	1824 (72.8)	**0.004**
**>5 years**	219 (32.2)	681 (27.2)	
**Father’s education**			
**≤5 years**	297 (26.5)	1120 (44.7)	0.164
**>5 years**	402 (29.0)	1385 (55.3)	
**Smoking in the residency**			
**Yes**	315 (31.4)	1004 (40.1)	**0.002**
**No**	384 (25.6)	1501 (59.9)	

**Table 2:** Sociodemographic characteristics of the students according to smoking status (continued variables).

	Smoker (n:699) mean±SD	Non smoker (n:1806) mean±SD	Total (n:2505) mean±SD	
**Age** **(min-max:17–46)**	21.26±2.60	20.72±2.44	20.87±2.50	<0.001
**BMI (Kg/m^2^)**	22.94±3.73	22.22±3.47	22.42±3.56	<0.001
**Number of family members**	5.76±1.92	5.91±1.96	5.87±1.95	0.068
**Number of siblings**	3.56±1.74	3.80±1.85	3.73±1.83	0.003

Comparison of categorical demographic characteristics according to smoking status showed that the smoking rate was higher in the following populations: men compared to women, alcohol consumers compared to non-users, having all close friends who smoked compared to some, having at least one smoker family member compared to none, studying in two-year faculties compared to four-year faculties, being in healthcare-related faculties compared to other faculties, having divorced parents (separated family) compared to married parents (intact family), having a mother with at least a secondary school education compared to lower levels, and the presence of smoking in their place of residency compared to absence (p s< .05; Table 1).

Comparison of continuous variables showed that, compared to non-smokers, smokers’ mean age was higher, mean body mass index was higher, mean family member number was lower, and mean number of siblings was lower (ps < .05; Table 2).

**Fig 1:**
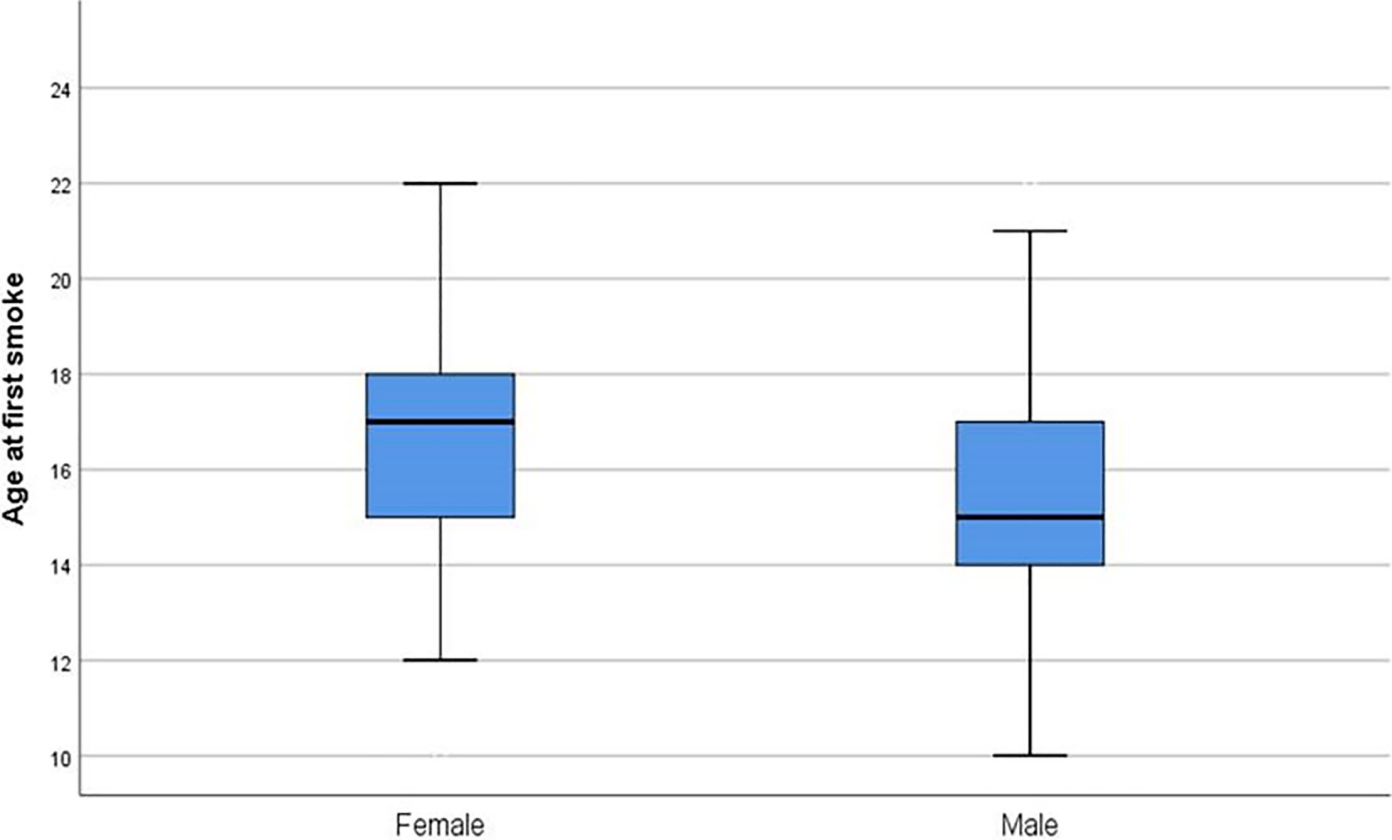
Age distribution of students’ first smoking experience per sex.

**Fig 2:**
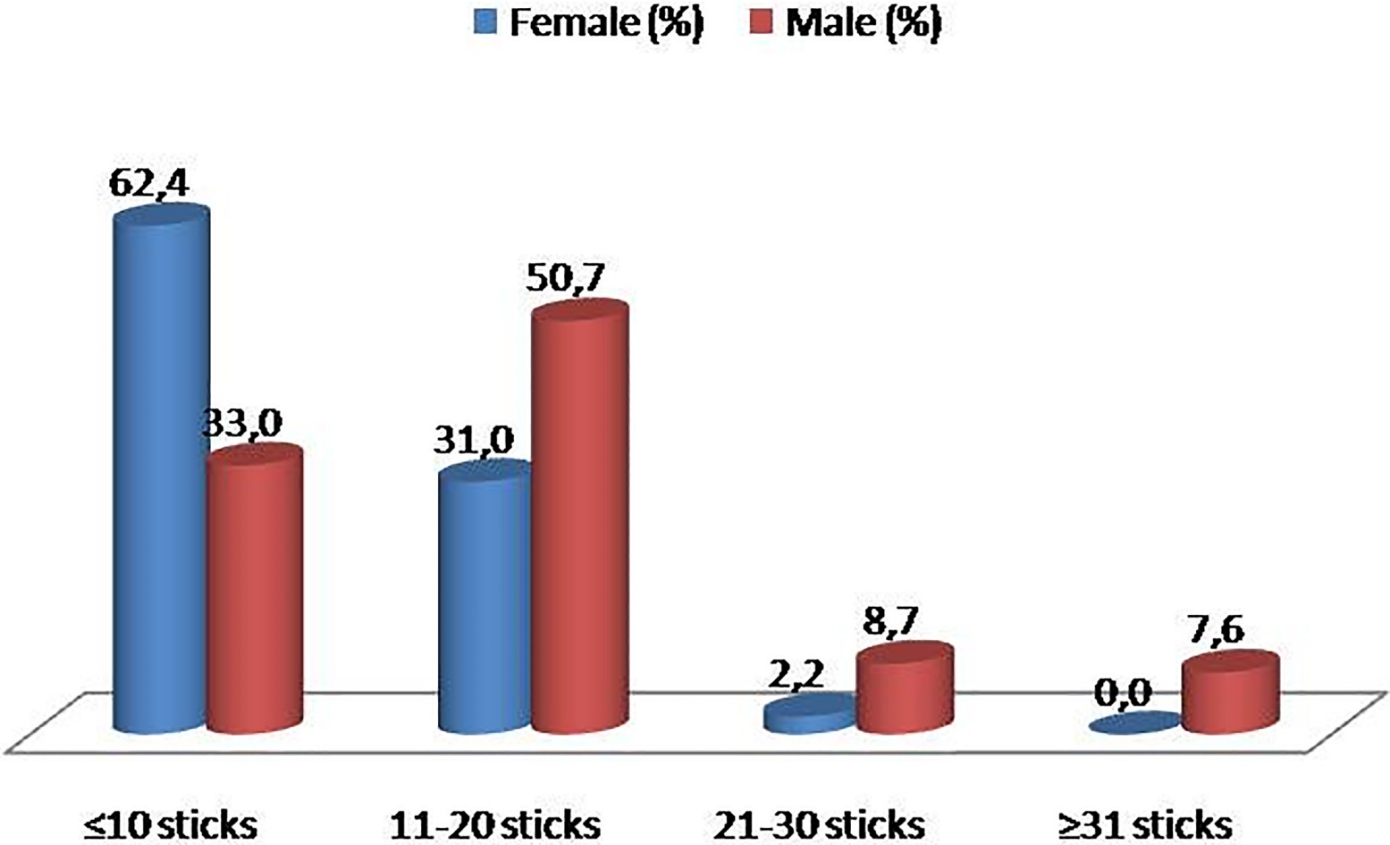
Students’ daily smoked cigarettes per sex.

**Fig 3:**
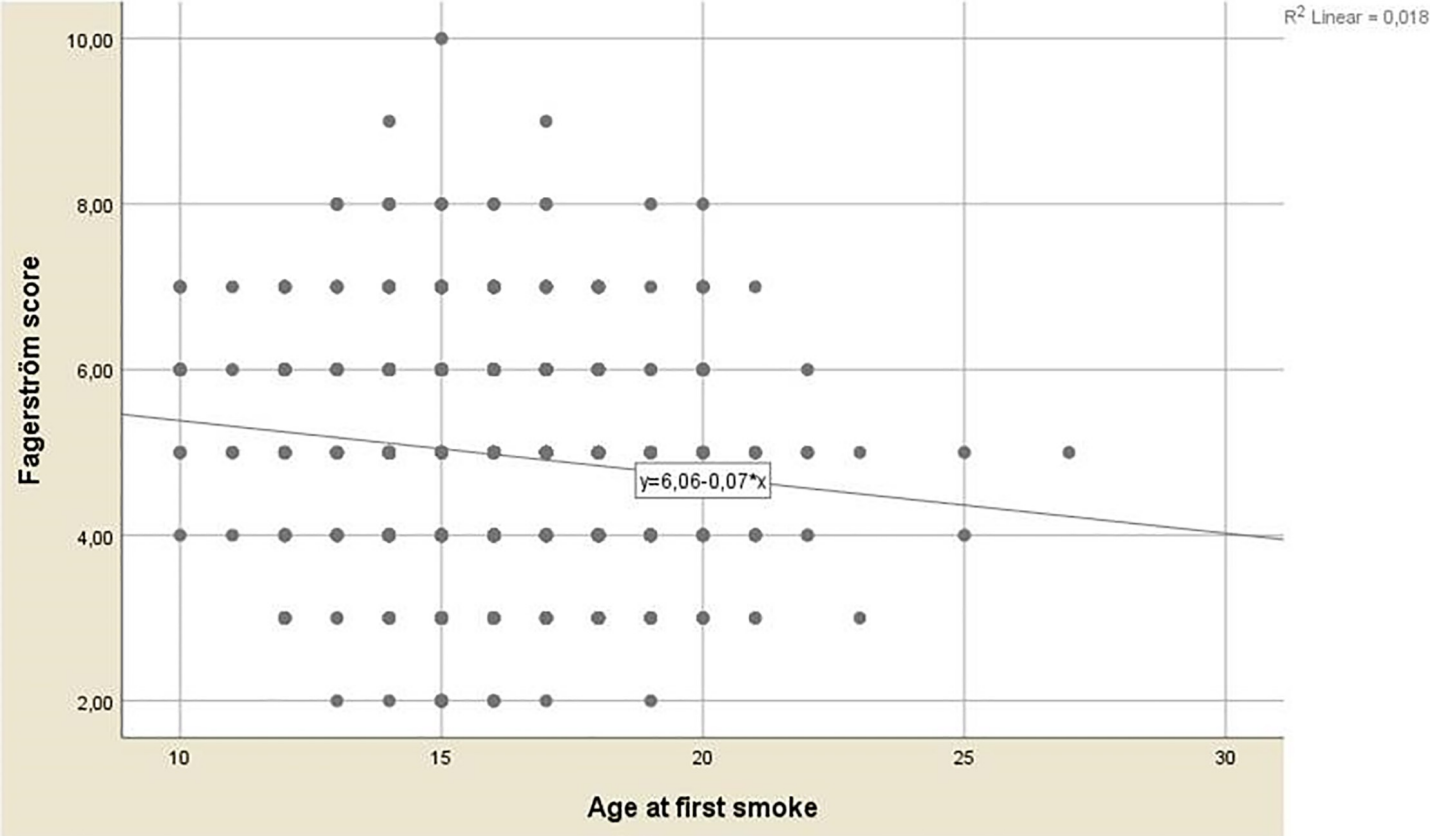
Correlation between smoking onset age and current smokers’ Fagerström scores.

Among smoker students, mean Fagerström test score was 4.74 ± 1.16 for women and 5.09 ± 1.31 for men (p < .05); mean age of starting smoking was lower in men compared to women (Fig 1). Additionally, men smoked 11 to 20 cigarettes per day on average, while females smoked less than 11 cigarettes daily (Fig 2). There was a negative correlation between nicotine dependency level and smoking onset age (r: -0.13, p < .001; Fig 3). Mean smoking onset age was 16.34 ± 2.72 years: 15.65 ± 2.67 years for men and 17.03 ± 2.60 years for women (p < .05; Fig 2).

When the effect of these variables on the entire study population was evaluated through multivariate logistic regression analyses, it was seen that being a man (odds ratio (OR): 3.43; 95% confidence interval (CI): 2.75–4.28), studying in two-year faculties (OR: 1.74; 95% CI: 1.39–2.18), having at least one close family member who smoked (OR: 1.63; 95% CI: 1.31–2.04), having all close friends who smoked (OR: 1.81; 95% CI: 1.40–2.33), and alcohol consumption (OR: 4.39; 95% CI: 3.51–5.49) were positively associated with current smoking (ps < .05; Table 3).

**Table 3:** Factors associated with smoking status in univariate and multivariate analysis.

	Univariate analysis			Multivariate analysis (Backward: LR)		
	OR	CI	p	OR	CI	p
**Age (Per 1 age increment)**	1.083	1.047–1.120	**<0.001**	1.037	0.995–1.082	0.088
**Sex (man v.s woman)**	4.705	3.901–5.675	**<0.001**	3.438	2.759–4.283	**<0.001**
**Curriculae (2 years faculty vs. 4 years faculty)**	2.187	1.830–2.613	**<0.001**	1.745	1.393–2.186	**<0.001**
**Faculty (other than healthcare vs. healthcare)**	1.469	1.189–1.814	**<0.001**	1.150	0.901–1.467	0.262
**BMI (Per 1 level increment)**	1.056	1.031–1.082	**<0.001**	0.988	0.959–1.018	0.415
**Academic level (Per 1 level increment)**	0.942	0.859–1.033	0.203	0.989	0.871–1.122	0.863
**Family type (Destroed vs. intact)**	2.428	1.491–3.953	**<0.001**	1.543	0.843–2.827	0.160
**Number of family member (Per 1 level increment)**	0.958	0.915–1.003	0.068	1.008	0.954–1.064	0.782
**Mother’s education level (>5 years vs. ≤5 years)**	1.117	1.047–1.192	**0.001**	1.050	0.817–1.351	0.701
**Father’s education level (>5 years vs. ≤5 years)**	1.082	1.016–1.153	**0.014**	1.123	0.894–1.410	0.320
**Smoking in the residency (Present vs. absent)**	1.330	1.115–1.587	**0.002**	1.206	0.976–1.491	0.083
**At least one smoker family member (Presence vs. absence)**	1.727	1.432–2.083	**<0.001**	1.639	1.314–2.044	**<0.001**
**Smoker close friends (all vs. some)**	3.777	3.046–4.683	**<0.001**	1.810	1.405–2.333	**<0.001**
**Alcohol consumption (Presence vs. absence)**	6.537	5.339–8.004	**<0.001**	4.391	3.511–5.491	**<0.001**

Note: 2 Loglikelihood: 2344.930, R2: 0.220 (Cox&Snell), 0.316 (Nagelkerke), omnibustest of MCchi-square: 621.221, p = 0.000

## Discussion

This study investigated the prevalence of cigarette smoking and the associated factors that would affect smoking behavior among students at Artvin Çoruh University. We showed that being a man, studying in two-year faculties, having all close friends who smoked, having at least one smoker family member, and consuming alcohol were positively associated with increased current smoking risks. Additionally, among daily smokers, men had higher nicotine dependency levels and a lower mean smoking onset age than did women. A negative correlation was found between Fagerström test score and smoking onset age.

According to data from the 2012 Global Adult Tobacco Survey, Turkey’s smoking rate was 27.1%: 41.5% among men and 13.1% among women [21], and this was similar to our results. According to the same survey, among those aged 15–24 years, the smoking rate was 33.0% in boys/men and 7.4% in girls/women. It is vital to monitor the younger population’s smoking status periodically. This study was the first to evaluate Artvin Çoruh University students’ smoking situation; therefore, it is not possible to make a comparison against the smoking status of the university students in previous years. However, it is possible to estimate general trends when comparing against other universities’ results in recent years.

According to current data, compared to the country’s overall cigarette smoking rate in that age group—and compared to university students’ smoking rates in studies conducted in last five years—our study population’s smoking levels were higher among both sexes [10, 21]. Previous studies conducted among Turkish students revealed that living in urban areas was a risk factor for smoking habits [22]. Some studies showed that increasing the cigarette tax discourages individuals from smoking [23, 24]; therefore, Turkey has followed MPOWER policies and increased the cost of cigarettes [3]. However, in eastern regions of the country, illicit cigarettes and other types of tobacco products are prominent [7]. The location of this study may have been a factor in students’ ability to obtain cheap/illicit cigarettes with minimal effort.

The high smoking rate in our study population can be further attributed to the ease of smoking and buying cigarettes on the university campus. In previous studies, it was shown that smoking bans on university campuses decreased the smoking rate [12, 25]; the university in the current study had no such ban. The lack of social and sport entertainment opportunities at the university may have also affected students’ smoking rates by contributing to students’ boredom. Therefore, to decrease smoking among this population and other university students, preventions such as smoking bans and offering student entertainment may be feasible and effective solutions.

Both univariate and multivariate logistic regression analyses showed that men were significantly more likely to smoke than were women. This result is similar to other findings both in Turkey [11] and globally [5, 18, 26]. We concur with previous studies [5, 26], that this might have been because of the internalization of “gender roles” by the participants, and because of the societal and cultural acceptance of smoking among men rather than women. Further, women’s daily cigarette use and their nicotine dependency were lower compared to men. One explanation for this distinction could be the higher mean smoking onset age of women—it has been previously reported that younger ages are associated with higher dependency and lower quitting rates [27, 28]. According to the 2012 Global Adult Tobacco Survey, the mean age for starting smoking was 19 years; however, novel data shows that this age is decreasing: 16 years on average [21]. Future studies would benefit from examining smoking rates per sex instead of overall rates.

In our study and others, alcohol consumption was associated with an increased risk of smoking [14]. Studies have shown that alcohol use is a risk factor for early initiation of cigarette smoking [29] and continuation of smoking [30]. Additionally, students in the two-year faculties (vs. four-year) had higher smoking rates. In Turkey, the only difference between two- and four-year faculties is that acceptance into four-year faculties requires higher university exam marks. This association between low academic success and smoking behavior has been previously studied, and low academic performance was found to be a predictor for smoking [31].

The strongest association was between smoking and having both close friends and parents who smoke, which reflects prior results [15, 18, 32]. The influence of parental smoking seems less clear; however, believing that family members smoke and having a positive attitude toward smoking were both previously noted factors that were predictive for smoking [33]. In a recent study, living in intact families was shown to be a predictive factor for never smoking among students [34]. In another study, living situation, mothers’ educational level, economic status, and parents’ marital status were found to be the most influential predictive factors for substance abuse, including smoking [35]. Other studies have shown that overall smoking prevalence increases with age; similarly, the comparison of first and last year students’ smoking prevalence in Turkey shows that prevalence is higher among last-year students compared to first-year students [9, 36]. In the current study, these results were only significant in the univariate analysis (not the multivariate analysis).

### Strengths and limitations

This study was unique in several ways that make it valuable to the literature on Turkish smoking habits, particularly among youths. The large sample size makes it possible to generalize the results among university students in this region more easily. Similarly, we evaluated both two- and four-year faculties—a gap in data that required bridging [37]. The location also allowed for data from a region that plays a role in the illicit transport of tobacco products into Turkey. Furthermore, there have not been any studies on university students’ smoking behavior in the last 5 years, meaning that our study adds new information to the data pool for this population. However, there are some limitations, including a lack of proof of illicit tobacco use and the cross-sectional design, which eliminates any temporal association. More studies from diverse regions in Turkey using both qualitative and quantitative measures are needed.

## Conclusions

In conclusion, this study evaluated the smoking status and the factors affecting smoking habits of university students located in a small city in Turkey. Our results showed higher smoking rates among students compared to the country’s general population in that age range; the data were also higher compared to other Turkish university students according to recent data. That result may be related to not only the geographical location of the study but also differences in students’ academic success. Even after considering these factors, the smoking rate of our study population is alarming. It is essential to review the government tobacco control policies to determine how effective they are for university students. Potential interventions could include campus-wide smoking bans, increased social/sport entertainment, and more educational activities about the health risks associated with smoking.

## Supporting information

S1 Questionaire English. (DOCX)

S2 Questionaire Turkish. (DOCX)

S1 Dataset Karadogan onal kanbay research dataset. (SAV)
