# Supplementary material for: Prevalence and determinants of smoking status among university students: Artvin Çoruh University sample
Source: PLoS One. 2018 Dec 10;13(12):e0200671. doi: 10.1371/journal.pone.0200671 (PMC6287842; doi:10.1371/journal.pone.0200671)
Supplement: S1 Questionaire — (DOCX) [file pone.0200671.s001.docx]

**DEAR PARTICIPANT;**

This study is conducted to evaluate ‘Prevalence and determinants of smoking status among university students’. Your participation will make it strong and will be important to give novel data to literature. Please do not write your name on questionnaire. Thank you for your collaboration.

**Communication:** Assistant. Prof. Dr. Yalçın KANBAY, Artvin Çoruh Üniversity Faculty of Health Sciences // [yalcinkanbay@hotmail.com](mailto:yalcinkanbay@hotmail.com)

**PERSONAL DATA FORM**

| **1. Gender:** ☐Female ☐Male  **2. Age:** ………  **3.** **Height:**……cm **Weight:**……Kg  **4. Faculty/School Name:…………………**  **5.2-year or 4-year section, please select:**….………………..  **6. Department:……….**  **7. Class :……….**  **8. Family type:**  ☐Cor family ☐Wide family ☐Seperated family  **9. Number of family members including you:……….**  **10. Number of siblings (including you):……….**  **11. Education level of your mother**  ☐Illiterate ☐Literate  ☐Primary school ☐Secondary school  ☐High school ☐2 years universiyt  ☐4 year university ☐Over 4 year university  **12. Education level of your mother**  ☐Illiterate ☐Literate  ☐Primary school ☐Secondary school  ☐High school ☐2 years university  ☐4 year university ☐Over 4 year university  **13. Did you ever smoked?**  ☐Yes ☐No  ***If yes, how old were you :…………………….***  **14. Have you smoked over 100 sticks of cigarettes**  **till that date?**  ☐Yes ☐No  **15. Over the past month did you smoke?**  ☐Yes ☐No  ***If yes, how many cigarettes did you smoke on average?*:………….** | **16. Are any of your close friends smoker?**  ☐None of them ☐Some of them ☐All of them  **17. Who is smoking in your family?**  ☐None ☐Mother ☐Father ☐Sibling  **18. Is it allowed to smoke cigarette at indoor/collective**  **areas where you live?**  ☐Yes ☐No  **19. What is your frequency of alcohol use?**  ☐I never drink ☐ I rarely drink  ☐I occationally drink ☐I often drink  **If you smoke regularly, answer the following questions. Otherwise, leave blank**  **1. How many cigarettes/day do you smoke?**  ☐10 or less ☐11-20  ☐21-30 ☐31 or more  **2. How soon after waking do you smoke your first**  **cigarette?**  ☐Within 5 minutes ☐6-30 dakika içinde  ☐31-60 minutes ☐After 60 minutes  **3. Do you find it difficult to refrain from smoking in**  **places where it is forbidden?**  ☐Yes ☐No    **4. Which cigarette would you hate most to give up?**  ☐The first one in the morning ☐All others  **5. Do you smoke more frequently during the first hours after waking than during the rest of the day?**  ☐Yes ☐No  **6. Do you smoke if you are so ill that you are in bed most of the day?**  ☐Yes ☐No |
| --- | --- |
