# Supplementary material for: Prevalence and determinants of smoking status among university students: Artvin Çoruh University sample
Source: PLoS One. 2018 Dec 10;13(12):e0200671. doi: 10.1371/journal.pone.0200671 (PMC6287842; doi:10.1371/journal.pone.0200671)
Supplement: S2 Questionaire — (DOCX) [file pone.0200671.s002.docx]

**DEĞERLİ KATILIMCI;**

Bu çalışma **“Üniversite Öğrencilerinin Sigara İçme Durumları ve Etkileyen Etmenlerin Belirlenmesi”** amacıyla planlanmıştır. Çalışmaya katılımınız planlanan araştırmanın sonuca ulaşması açısından oldukça önem arz etmekte olup bilime önemli katkı sağlamış olacaksınız. Soruları içtenlikle doldurabilirsiniz. Soru formuna isminizi kesinlikle yazmayınız. Gösterdiğiniz destek ve anlayış için teşekkür ederiz.

**İletişim:** Yrd. Doç. Dr. Yalçın KANBAY, Artvin Çoruh Üniversitesi Sağlık Bilimleri Fakültesi // [yalcinkanbay@hotmail.com](mailto:yalcinkanbay@hotmail.com)

**KİŞİSEL TANITIM FORMU**

| **1. Cinsiyet:** ☐Kadın ☐Erkek  **2. Yaş:** ………  **3.** **Boy:**………….cm **Kilo:**………Kg  **4. Fakülte/YO/**  **5. İki yıllık veya 4 yıllık bölümlerden hangisi :**….………………..  **6. Bölüm:……….**  **7. Sınıf:……….**  **8. Aile tipiniz:**  ☐Çekirdek aile ☐Geniş aile ☐Dağılmış aile  **9. Siz dahil ailenizdeki birey sayısı:……….**  **10. Kardeş sayınız (siz dahil):……….**  **11. Annenizin Eğitim Durumu**  ☐Okur- yazar değil ☐Okur- yazar  ☐İlkokul ☐Ortaokul  ☐Lise ☐Önlisans  ☐Lisans ☐Lisansüstü  **12. Babanızın Eğitim Durumu**  ☐Okur- yazar değil ☐Okur- yazar  ☐İlkokul ☐Ortaokul  ☐Lise ☐Önlisans  ☐Lisans ☐Lisansüstü  **13. Bu güne kadar hiç sigara denediğiniz oldu mu?**  ☐Evet ☐Hayır  ***Cevabınız evet ise kaç yaşında:…………………….***  **14. Bu güne kadar içtiğiniz toplam sigara adedi 100**  **Adet üzerinde midir?**  ☐Evet ☐Hayır  **15. Son bir aydır sigara içtiğiniz oldu mu?**  ☐Evet ☐Hayır  ***Cevabınız evet ise günde ortalama kaç adet içtiniz*:………….** | **16. Yakın arkadaşlarınızdan sigara içen var mı?**  ☐Hiçbiri ☐Bazıları ☐Hepsi  **17. Ailenizde kimler sigara içiyor?**  ☐Hiçbiri ☐Anne ☐Baba ☐Kardeş  **18. Ev içerisi gibi kapalı / toplu yaşadığınız alanlarda**  **sigara içiliyor mu?**  ☐Evet ☐Hayır  **19. Alkol kullanma sıklığınız?**  ☐Hiç içmem ☐Nadiren içerim  ☐Ara sıra içerim ☐Sık sık içerim  **Düzenli olarak sigara kullanıyorsanız aşağıdaki soruları cevaplayınız. Aksi halde boş bırakınız**  **1. Her gün genellikle kaç sigara içiyorsunuz?**  ☐10 veya daha az ☐11-20  ☐21-30 ☐31 veya daha fazla  **2. İlk sigaranızı uyandıktan ne kadar sonra içiyorsunuz?**  ☐5 dakika içinde ☐6-30 dakika içinde  ☐31-60 dakika içinde ☐60 dakikadan sonra  **3. Sigara içilmeyen yerlerde sigara içmemekte zorlanıyor musunuz?**  ☐Evet ☐Hayır    **4. Hangi sigaradan vazgeçmekte en çok zorlanırsınız?**  ☐Sabah ilk içilen ☐Diğer  **5. Günün ilk saatlerinde sonraki saatlere göre daha sık**  **sigara içiyor musunuz?**  ☐Evet ☐Hayır  **6. Çok hasta olduğunuzda veya günün çoğunu yatakta geçirdiğinizde sigara içer misiniz?**  ☐Hayır ☐Evet |
| --- | --- |
